# O01 Rapid respiratory microbiological POCTs and antibiotic use in primary care: the RAPID-TEST RCT

**DOI:** 10.1093/jacamr/dlae217.001

**Published:** 2025-01-23

**Authors:** Alastair Hay, Samantha Abbs, Matthew Ridd, Stephen Granier, Athene Lane, Peter Muir, Jodi Taylor, Grace Young, Kathy Eastwood, Hayly Dash, Lynne Bradshaw, Rebecca Clarke, Mandy Lui, Emma Bridgeman, Rachel Brierley, Emily Brown, Hannah Thornton, Lucy Yardley, Chris Metcalfe

**Affiliations:** University of Bristol, Bristol, UK; University of Bristol, Bristol, UK; University of Bristol, Bristol, UK; University of Bristol, Bristol, UK; University of Bristol, Bristol, UK; University of Bristol, Bristol, UK; University of Bristol, Bristol, UK; University of Bristol, Bristol, UK; University of Bristol, Bristol, UK; University of Bristol, Bristol, UK; University of Bristol, Bristol, UK; University of Bristol, Bristol, UK; University of Bristol, Bristol, UK; University of Bristol, Bristol, UK; University of Bristol, Bristol, UK; University of Bristol, Bristol, UK; University of Bristol, Bristol, UK; University of Bristol, Bristol, UK; University of Bristol, Bristol, UK

## Abstract

**Background:**

Antibiotics are prescribed for over 50% of RTIs in primary care, despite good evidence most patients do not benefit. Rapid multiplex microbiological point-of-care-tests (POCTRM) determine the presence/ absence of respiratory pathogens in 15 min. Whether POCTRM reduce antibiotic prescribing without worsening patient outcomes is not known.

**Objectives:**

To investigate whether POCTRM can (i) reduce same-day antibiotic prescribing for children and adults presenting to primary care with RTIs; and (ii) change symptom severity on Days 2 to 4.

**Methods:**

Patients 12+ months with a RTI where the clinician and/or patient believed antibiotic treatment was, or may have been necessary, presenting to 16 GP practices in SW England. Patients individually randomized to BioFire^®^ FilmArray^®^ Torch 1 testing for 19 viruses and four atypical bacteria; or usual care.

**Results:**

Recruitment opened December 2022 and ended April 2024. A total of 552 participants were randomized: 63% female; 86% aged 16+ years; 95% self-reported white ethnicity; 26% had chronic lung disease; 5% reported a temperature >38°C; 14% reported tachycardia (age-adjusted); 8% reported tachypnoea (age-adjusted); 1% had oxygen saturation <94%. Ninety-five percent provided a dual throat and nasal swab, and clinician diagnoses included: acute cough (17%); common cold (14%); acute pharyngitis/ tonsillitis (13%); acute lower RTI (11%); chest infection (9%); sore throat (9%); and infective exacerbation of chronic lung disease (7%). Outcome data completeness: 548 (99%) with antibiotic prescribing; 414 (76%) with Day 2 to 4 symptom severity.

**Conclusions:**

Full results will be available for conference. This globally original RCT will provide important evidence regarding the role of rapid multiplex microbiological point-of-care-tests in primary care.

